# Assessing Cognitive Function in Multiple Sclerosis With Digital Tools: Observational Study

**DOI:** 10.2196/25748

**Published:** 2021-12-30

**Authors:** Wan-Yu Hsu, William Rowles, Joaquin A Anguera, Annika Anderson, Jessica W Younger, Samuel Friedman, Adam Gazzaley, Riley Bove

**Affiliations:** 1 Department of Neurology Weill Institute for Neurosciences University of California San Francisco, CA United States; 2 Neuroscape University of California San Francisco, CA United States; 3 Department of Psychiatry University of California San Francisco, CA United States; 4 Department of Physiology University of California San Francisco, CA United States

**Keywords:** cognition, digital health, mHealth, multiple sclerosis, cognitive assessment

## Abstract

**Background:**

Cognitive impairment (CI) is one of the most prevalent symptoms of multiple sclerosis (MS). However, it is difficult to include cognitive assessment as part of MS standard care since the comprehensive neuropsychological examinations are usually time-consuming and extensive.

**Objective:**

To improve access to CI assessment, we evaluated the feasibility and potential assessment sensitivity of a tablet-based cognitive battery in patients with MS.

**Methods:**

In total, 53 participants with MS (24 [45%] with CI and 29 [55%] without CI) and 24 non-MS participants were assessed with a tablet-based cognitive battery (Adaptive Cognitive Evaluation [ACE]) and standard cognitive measures, including the Symbol Digit Modalities Test (SDMT) and the Paced Auditory Serial Addition Test (PASAT). Associations between performance in ACE and the SDMT/PASAT were explored, with group comparisons to evaluate whether ACE modules can capture group-level differences.

**Results:**

Correlations between performance in ACE and the SDMT (R=–0.57, *P*<.001), as well as PASAT (R=–0.39, *P*=.01), were observed. Compared to non-MS and non-CI MS groups, the CI MS group showed a slower reaction time (CI MS vs non-MS: *P*<.001; CI MS vs non-CI MS: *P*=.004) and a higher attention cost (CI MS vs non-MS: *P*=.02; CI MS vs non-CI MS: *P*<.001).

**Conclusions:**

These results provide preliminary evidence that ACE, a tablet-based cognitive assessment battery, provides modules that could potentially serve as a digital cognitive assessment for people with MS.

**Trial Registration:**

ClinicalTrials.gov NCT03569618; https://clinicaltrials.gov/ct2/show/NCT03569618

## Introduction

### Background

Multiple sclerosis (MS) is a chronic inflammatory and neurodegenerative disorder, and it is the leading cause of major disability in young adults. Cognitive impairment (CI) occurs in 30%-70% of patients with MS [1,2], even in the absence of physical impairment [1,3,4]. CI is one of the most debilitating manifestations of MS and can have a profound influence on a patient’s personal independence and quality of life, interfering with social functioning and employment. Since 2014, CI assessment has been one of the measure specifications of the American Academy of Neurology’s MS Quality Measurement Set [5]. Ideally, patients with MS should undergo a complete cognitive assessment and routinely repeat the examination to detect cognitive changes overtime and to start timely treatment, if needed. However, to date, cognitive assessment in MS relies on a comprehensive neuropsychological examination, which is time-consuming and extensive; therefore, it makes it difficult to include cognitive assessment/monitoring as part of MS standard care.

### Cognitive Assessment with Digital Tools

Integrating digital tools into clinical settings can reduce the time and cost associated with cognitive examination and further allows repeated assessments, which provide more precise monitoring of cognitive performance and longitudinal changes. One more feature of digital tools is the capability of remote and self-administration, which can relieve the burden of travel to the clinic, due to deficits in mobility or cognition for many patients. Moreover, with remote administration features, data collection can be performed in a nonclinical, real-life setting, which allows sampling of cognitive performance more closely reflecting real-world cognitive function [6]. The self-administered feature also reduces common stressors for patients who get nervous during structured testing in clinical settings.

With advanced technology, remote, computerized platforms for cognitive assessment and treatment, using personalizing features, including adaptive staircase algorithms for populations with cognitive deficits [7-14], have been developed. In Alzheimer’s disease–related dementias, digital cognitive assessment tools have shown reliability in measuring longitudinal cognitive changes in individuals with no CI, mild CI, and dementia [15,16] and have exhibited cross-sectional sensitivity to cerebrospinal fluid amyloid-ß levels [17]. In schizophrenia, digital assessments have also shown effectiveness in identifying deficits across different cognitive domains [18,19]. Moreover, tablet-based cognitive assessment has been validated to differentiate cognitive control ability between children with and without 16p11.2 deletion, a genetic variation implicated in attention deficit/hyperactivity disorder and autism [8]. Although the development and validation of digital cognitive assessment tools have been growing, the investigations of remote, digital CI assessments in MS have been scarce [20-23]; therefore, there is a need to deepen the exploration of digital cognitive evaluation for improving access to CI assessments in order to thereby navigate problems related to cognitive issues and further reduce the impact of CI on patients’ lives.

### Aims and Overview of the Study

The goal of this study was to evaluate the feasibility and potential assessment sensitivity of a tablet-based cognitive assessment battery in patients with MS, focusing on the most commonly affected cognitive domains in MS: processing speed, attention, executive function, and memory [1,2]. To accomplish this, a tablet-based cognitive assessment battery (Adaptive Cognitive Evaluation [ACE]; see the Methods section) that measures different aspects of high-order cognitive function (eg, attention, working memory, speed of information processing, and executive function) [24], was tested in 53 participants with MS and 24 participants without MS. ACE was developed by Neuroscape at the University of California, San Francisco (UCSF) [24]. It has a user-friendly interface as well as adaptive algorithms, which modulate the challenge level of a task on a trial-by-trial basis based on individual performance. In this study, 3 modules (Boxed, Sustained Attention ACE Task [SAAT], Spatial Span) assessing different aspects of cognitive control ability and 1 module (Basic Reaction Time [BRT]) measuring the basic response speed were included (see the Methods section) for a preliminary examination of the construct validity of the ACE battery. The Symbol Digit Modalities Test (SDMT), a test considered the most sensitive measurement for the evaluation of cognitive involvement and information processing speed in the early MS course [25,26], was also administered. Given that information processing speed has been shown to account for impairments in high-level cognitive functions in MS [27-30], the relationship between performance in the SDMT and ACE modules was investigated. To further delineate whether ACE modules can differentiate different levels of cognitive function, performance differences among participants with MS with and without CI, as well as participants without MS, were examined.

We hypothesized that there would be a correlation between the SDMT score and ACE performance in accordance with the relative consequence theory of information processing speed [30-32], in which impaired processing speed is considered the key deficit underlying CI in MS [27-30]. In addition, as a tool for cognitive assessment in MS, ACE would reveal group-level differences between participants with MS with and without CI, as well as participants without MS.

## Methods

### Participants

In total, 53 adults with clinically definite MS [33], mean age 51.8 (1.7) years, were recruited from the University of California, San Francisco Multiple Sclerosis and Neuroinflammation Center between April 2018 and January 2019 with the following inclusion criteria: internet connection available at home or in the work environment and free of relapses or steroid use in the past month. Patients with severe visual, cognitive, or motor impairment that would preclude the use of a tablet-based tool were excluded. A group of 24 adults without MS (non-MS), mean age 46.0 (3.7) years, with no chronic autoimmune diseases were also recruited from the UCSF staff, willing family members of patients in the clinic, and other eligible and willing volunteers.

### Standard Approval, Registration, and Patient Consent

All procedures performed in the study involving human participants were approved by the Committee for Human Research at the UCSF. Written informed consent was obtained from each participant. The trial is registered with clinicaltrials.gov (NCT03569618).

### Study Design

To evaluate ACE, both ACE and the SDMT were administered to all participants, including 53 adults with MS and 24 adults without MS (non-MS). All participants with MS were recruited as part of studies to determine the feasibility [20] and preliminary efficacy [34] of a digital cognitive treatment, as previously described [34]. As part of a published study in which another tablet-based assessment (ie, EVO Monitor) was investigated [35], this study contains data that have not been analyzed or published in a larger trial (clinicaltrials.gov NCT03569618). The analysis of this study was based on baseline performance data (ie, before any cognitive intervention) of our feasibility [20] and efficacy [34] trials, where participants underwent cognitive testing, including ACE and the SDMT. The 2-hour baseline session began with standard measures (SDMT, the Paced Auditory Serial Addition Test [PASAT], the California Verbal Learning Test Second Edition, and the Brief Visuospatial Memory Test Revised), followed by digital cognitive assessment with ACE (BRT, Boxed, SAAT, Spatial Span) and EVO Monitor (data presented in [35]). Standard measures were administered by a study coordinator. Digital tool assessment was self-guided; however, a study coordinator sat in with the participants to answer questions and clarify aspects of the directions, if needed. The baseline session did not include any predetermined break, while participants were informed at the beginning of the visit that they could take a break at any time, if needed. Task order was predetermined (as described above) and remained consistent through the whole study. Only the SDMT and PASAT were included in the analysis of this study, given that they are the most widely used standard measures for people with MS and the cognitive domains being evaluated by these tests are close to cognitive aspects that ACE is designed to test for (ie, attention, working memory, speed of information processing, and executive function).

### Cognitive Measures

#### Standard Measures: SDMT and PASAT

SDMT is a widely used measure of selective attention and information processing speed in MS [25,26], which requires the participant to substitute geometric symbols for numbers while scanning a response key. The participants were presented with a page headed by a key that pairs 9 symbols with the single digits 1-9. Rows below showed only symbols, and the task was to write the correct number in the spaces below based on the key row. After finishing the first 10 items with guidance, correct responses being made within 90 seconds were counted as the SDMT score.

Since PASAT has also been used extensively to test information processing speed, attention, and working memory in MS [36], we included it as a standard measure for participants with MS recruited in this study. The participants were instructed to listen to numbers presented every 3 seconds and add the number they hear to the number they heard before (rather than giving a running total). The PASAT score was defined as the total number of correct answers out of 60 possible answers.

#### Digital Cognitive Assessment Battery: ACE

**Figure 1:**
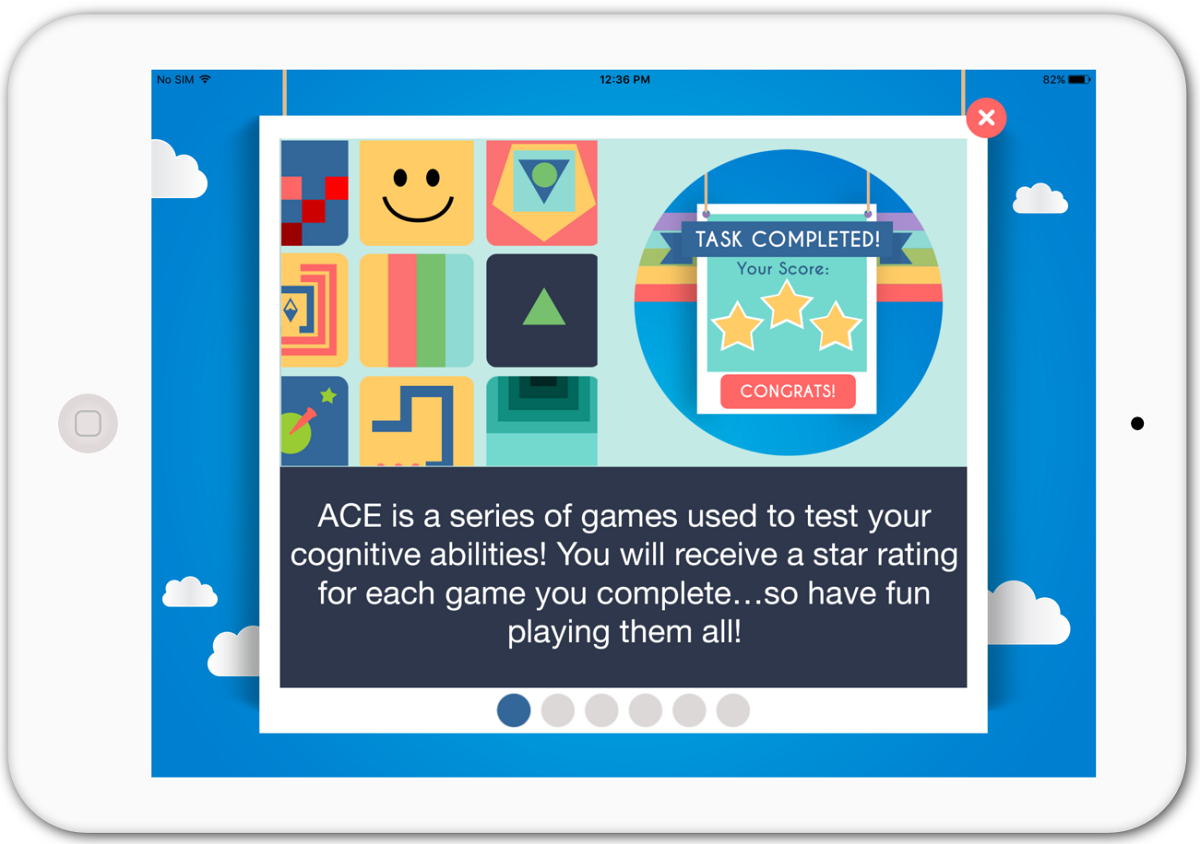
Screenshot of ACE. ACE: adaptive cognitive evaluation.

Tasks within ACE (Figure 1) followed a similar schematic: across modules, the probe or target (as specified in the individual module description below) was displayed either until a response was made or until the maximum reaction time (RT) limit was reached. After each trial, the trial-level feedback, either a green (correct response was made within the RT limit), yellow (correct response was made outside of the RT limit), or red (incorrect response) centralized fixation cross was displayed for 200 ms, followed by a standard 1000 ms intertrial interval.

The *BRT* task was designed to index the basic response speed of participants on a simple task with minimal loading on executive function skills [37]. Participants were instructed to press a button at the bottom of the screen as fast as they could when they detected a symbol (target) that appeared in the center of the screen. The target always appeared without distraction. The BRTs were measured for both index fingers. Participants first completed 5 practice trials for each of their right and left index fingers, followed by 20 experimental trials per index finger. This task started with a maximum RT limit of 500 ms and a response window of 500 ms that adapted for each participant according to trialwise performance. An average RT was measured for each hand. Only data from the dominant hand were included as each participant’s BRT in the following analyses.

The *Boxed* task was designed to measure visual search performance across different types and number of distractors [38]. Participants were presented with an array of either 4 or 12 Landolt squares (ie, squares with gaps) with an opening on 1 side until participants located the target (a green box with a gap on the top or bottom) and indicated the location of the gap (top or bottom) by tapping with their dominant hand on a button with either “top” or “bottom.” Two distinct search modes were included: a feature search, where red Landolt squares were present in addition to the single green target, allowing the target to be located based solely on object color, and a conjunction search, where distractor boxes were green and red, with all green distractor boxes having gaps on either side and red distractor boxes having gaps on the top and bottom, similar to the green target box. As such, participants had to search the array for the target square based on a conjunction of features: both color and position of the opening. In addition to the 2 search types (feature and conjunction), there were 2 distractor load conditions: a low load with 1 target and 3 distractors and a high load with 1 target and 11 distractors. Participants completed 8 practice trials of each condition (32 total) before moving on to the experimental task with 25 trials per condition (100 total). This task started with a maximum RT limit of 1500 ms and a response window of 1000 ms that adapted for each participant according to trialwise performance. Only correct responses made within the participants’ adaptive response window were included in analysis. Task performance was assessed by examining the mean RT to correct responses for all trial types, including 4- and 12-item trials collapsed across both feature and conjunction search modes. The RT cost between target identification for feature and conjunction trials across each set size was measured as distraction cost = 12-item (conjunction and feature) – 4-item (conjunction and feature).

*SAAT* was developed based on the Tests of Variables of Attention (TOVA) [39] and was designed to include blocks that separately measure sustained attention (to an infrequent target) and inhibitory control (inhibiting a prepotent response to salient distractors). During a trial, a symbol (target) appeared at the top or bottom of the screen. Participants were instructed to press a button with the index finger of only their dominant hand when the target appeared at the top of the screen and to ignore the symbol and withhold a response when it appeared at the bottom. This task proceeded in 2 blocks. In the inhibitory control block, targets appeared on 27 of 40 (67%) of trials and required participants to withhold a (highly primed) response when a distractor appeared. In the sustained attention block, the target appeared on only 13 of 40 (33%) of trials, requiring participants to maintain attention to avoid missing an infrequent target. Participants completed 10 practice trials (6 target and 4 nontarget trials) and then 80 experimental trials, 40 in each block. This task started with a maximum RT limit of 600 ms and a response window of 600 ms that adapted for each participant according to trialwise performance. However, to avoid creating an artificially low response window, correct rejections did not affect the response window. Trials where no response was given (when a response was expected) or that were anticipatory (RT < 150 ms) were excluded from analyses. All remaining trials were evaluated for accuracy regardless of whether the response was within the response window. Thus, trials were only considered incorrect if an incorrect response was made (and not if they were correct but late). The mean RT to correct responses collapsed across block types were measured as task performance.

The *Spatial Span Task* is a computerized version of the Corsi Block-Tapping Test [40], which has frequently been used to assess visuospatial working memory capacity. On each trial, participants viewed a test array of 20 black circles that were cued sequentially in line with the typical administration of the Corsi Block-Tapping Task stimuli. Cued circles were lit in green, one at a time, sequentially. After the sequence of circles was complete and no longer displayed, participants were instructed to recall the location of each cued circle in the order they were shown and indicate the location and sequence by tapping each cued location in the cued order. Participants started with between 2 and 4 practice trials with 3-location sequences (ie, 3 cued circles). Participants practiced until 2 consecutive trials were answered incorrectly. Regardless of practice performance, participants then began the experimental task with a 3-location sequence. Once the participant completed 2 consecutive trials of the previous level without an error, they would advance to the next level that included an additional cued circle, increasing the difficulty level. Participants completed as many levels as possible until 2 consecutive incorrect trials, at which point the task ended. Participants had unlimited time to respond for this task. Participants needed to have successfully completed at least 2 3-location sequence trials to be included in analysis. The highest level (ie, maximum number of items in a sequence) of the successful trial for each participant was defined as the spatial span, the measurement of working memory capacity.

### Statistical Analysis

All numerical data are presented as the mean (SE). To evaluate the digital cognitive assessment battery (ie, ACE), Pearson correlation analyses were conducted to scrutinize the relationship between performance in the SDMT/PASAT and ACE modules. Partial correlation analyses with age, sex, years of education, and the BRT as covariates were applied, when appropriate. To examine whether the selected ACE modules can differentiate CI and non-CI participants with MS, participants with MS were divided into 2 subgroups (ie, CI and non-CI) according to their baseline SDMT z scores. Participants with an SDMT z score of <–1 based on published normative data [41] were characterized as CI. Differences between CI and non-CI participants with MS, as well as non-MS participants in terms of ACE performance, were examined by one-way ANOVA, with the BRT as a covariate to control for potential motor speed deficit in participants with MS. Two-tailed Student *t* tests were carried out for post hoc comparisons, when appropriate. The statistical significance threshold was set as *P*≦.05.

## Results

### Participants

**Figure 2:**
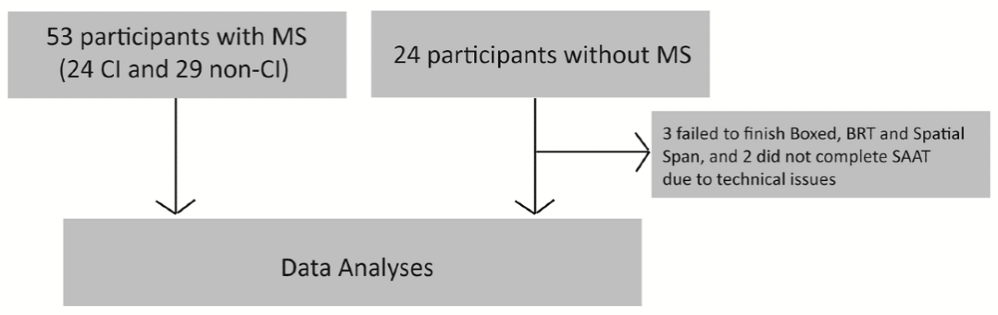
Task completion rate. ACE: Adaptive Cognitive Evaluation; BRT: basic reaction time; CI: cognitive impairment; MS: multiple sclerosis; SAAT: Sustained Attention ACE Task.

A total of 53 participants with MS (mean age 51.8 [1.7] years) and 24 participants without MS (mean age 46.0 [3.7] years) completed the assessments; their demographic and clinical characteristics are summarized in Table 1. One-way ANOVA revealed no age differences between the groups (F(2,76)=1.42, *P*=.24). For categorical variables (ie, sex and race), chi-square tests showed no statistically significant association between groups and sex (X^2^(2)=5.31, *P*=.07) as well as race (X^2^(4)=2.90, *P*=.59). For analysis purposes, the 53 participants with MS were divided into CI (n=24 [45%]) and non-CI (n=29 [55%]) subgroups based on to their baseline SDMT z score. Figure 2 details the task completion rate.

**Table 1:** Demographic and clinical characteristics of participants.

Characteristics		MS^a^			Non-MS (n=24)
		CI^b^ (n=24)	Non-CI (n=29)		
	Age (years), mean (SE)	50.87 (2.51)	52.68 (2.35)	46.04 (3.72)	
	Sex (female), n (%)	17 (70)	23 (79)	12 (50)	
	Education (years), mean (SE)	16.50 (0.47)	16.79 (0.51)	16.16 (0.41)	
	Right-handedness, n (%)	22 (91)	23 (80)	50 (100)	
	Part- or full-time employed, n (%)	11 (45%)	15 (51%)	17 (70%)	
	SDMT^c^ score, mean (SE)	34.79 (1.34)	49.34 (1.12)^d^	51.20 (2.65)^e^	
	SDMT z score, mean (SE)	–1.58 (0.08)	–0.05 (0.10)^d^	0.26 (0.20)^e^	
	Expanded Disability Status Scale (EDSS), median (IQR)	4 (1.75)	3 (1.5)	N/A^f^	
	Disease duration (years), mean (SE)	11.95 (1.83)	13.71 (1.49)	N/A	
**Race, n (%)**					
	White	22 (92%)	22 (76%)	19 (79%)	
	Black/African American	N/A	2 (7%)	1 (4%)	
	Other/unknown	2 (8%)	5 (17%)	4 (17%)	
**MS subtype, n (%)**					
	Relapsing-remitting	18 (75%)	23 (79%)	N/A	
	Primary progressive	2 (8%)	2 (7%)	N/A	
	Secondary progressive	3 (13%)	3 (10%)	N/A	
	Clinically isolated syndrome (CIS)	N/A	1 (3%)	N/A	
	Unknown	1 (4%)	N/A	N/A	

^a^MS: multiple sclerosis.

^b^CI: cognitive impairment.

^c^SDMT: Symbol Digit Modalities Test.

^d^*P*<.001 for the comparison between CI and non-CI groups.

^e^*P*<.001 for the comparison between CI and non-MS groups.

^f^N/A: not applicable.

### Correlation Between Standard Measures and ACE

**Figure 3:**
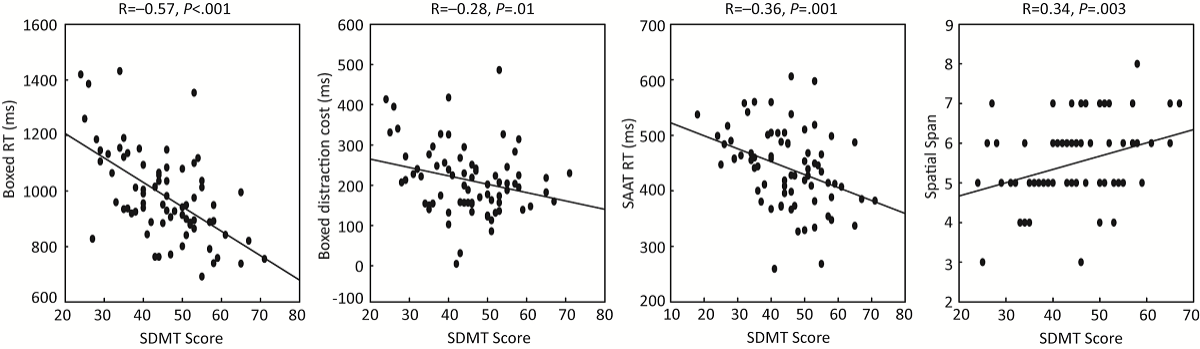
Correlation between SDMT score and performance in ACE modules. ACE: Adaptive Cognitive Evaluation; RT: reaction time; SAAT: Sustained Attention ACE Task; SDMT: Symbol Digit Modalities Test.

To delineate associations between performance in standard measures (ie, SDMT and PASAT scores) and the tested digital cognitive platform (ie, ACE), Pearson correlation analyses were performed. The SDMT showed significant correlations with several ACE measures (Boxed RT: R=–0.57, *P*<.001; Boxed distraction cost: R=–0.28, *P*=.02; SAAT RT: R=–0.36, *P*=.001; Spatial Span: R=0.34, *P*=.003; Figure 3). Since the Boxed RT showed the strongest correlation with the SDMT, we further performed an exploratory linear regression analysis to examine to what extent the Boxed RT value can be used to predict the SDMT score. The analysis revealed a moderate R-squared value of 0.333 with the regression equation SDMT = 82.55 – 0.038 × Boxed RT; 33% of the total variation in the SDMT score can be explained by the Boxed RT.

When controlling for age, sex, years of education, and the BRT with partial correlations, similar results were observed (Boxed RT: R=–0.44, *P*<.001; Boxed distraction cost: R=–0.28, *P*=.01; SAAT RT: R=–0.17, *P*=.15; Spatial Span: R=0.18, *P*=.12; Table 2). When we restricted the analyses to only participants with MS, SDMT correlations with the Boxed RT and Boxed distraction cost remained statistically significant (Boxed RT: R=–0.50, *P*<.001; Boxed distraction cost: R=–0.34, *P*=.01; SAAT RT: R=–0.22, *P*=.10; Spatial Span: R=0.24, *P*=.07). Again, in adjusted correlation analyses, we saw similar results (Boxed RT: R=–0.43, *P*=.002; Boxed distraction cost: R=–0.38, *P*=.01; SAAT RT: R=–0.16, *P*=.26; Spatial Span: R=0.18, *P*=.21; Table 2).

**Figure 4:**
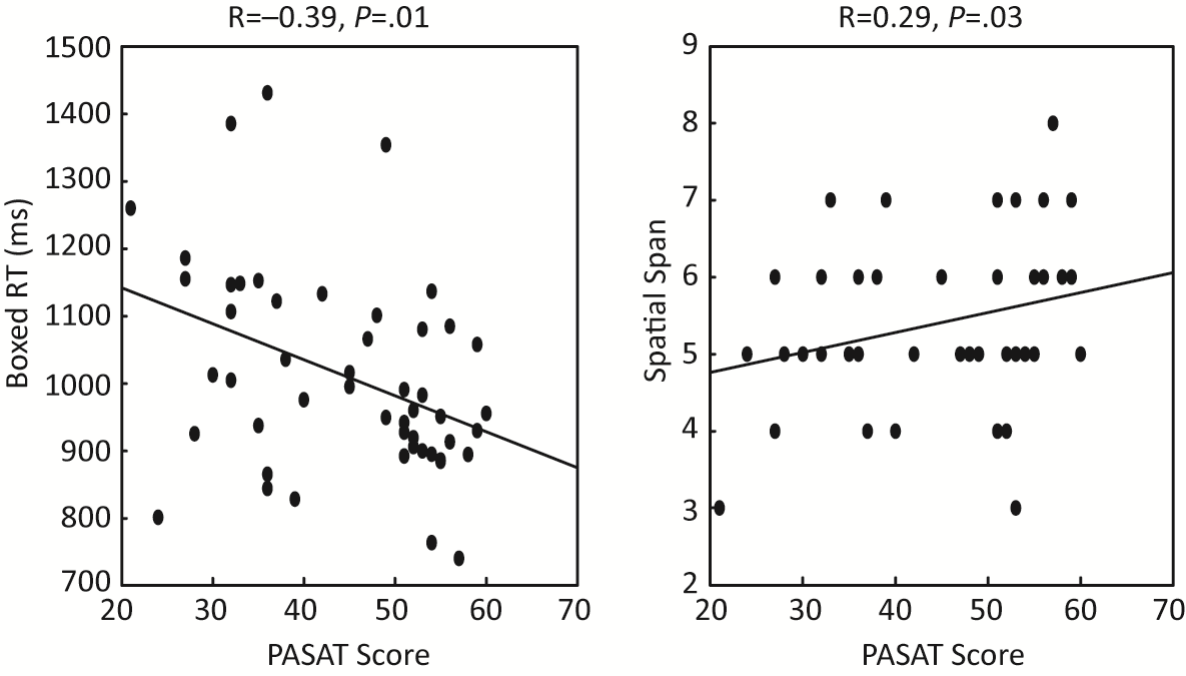
Correlation between PASAT score and performance in ACE modules. ACE: Adaptive Cognitive Evaluation; PASAT: Paced Auditory Serial Addition Test; RT: reaction time.

*PASAT*, which has also been extensively used to test cognitive function in MS, was tested in the participants with MS and also showed significant correlations with the Boxed RT (R=–0.39, *P*=.01) and Spatial Span (R=0.29, *P*=.03; Figure 4 and Table 3). These correlations remained significant after accounting for age, sex, years of education, and the BRT as covariates (Boxed RT: R=–0.40, *P*=.01; Spatial Span: R=0.34, *P*=.02). These results support the hypothesis that there is a correlational association between standard MS information processing measures and ACE measures.

**Table 2:** Results of Pearson correlation analyses between SDMT^a^ and ACE^b^ measures.

ACE measures			Covariates	R	*P* value
**All participants (N=77)**					
	Boxed RT^c^	N/A^d^		–0.57	*<.001* ^e^
	Boxed distraction cost	N/A		–0.28	*.01* ^e^
	SAAT^f^ RT	N/A		–0.36	*.001* ^e^
	Spatial Span	N/A		0.34	*.003* ^e^
	Boxed RT	age, sex, edu^g^, and BRT^h^		–0.44	*<.001* ^e^
	Boxed distraction cost	age, sex, edu, and BRT		–0.28	*.01* ^e^
	SAAT RT	age, sex, edu, and BRT		–0.17	.15
	Spatial Span	age, sex, edu, and BRT		0.18	.12
**Participants with MS^i^** **(N=53)**					
	Boxed RT	N/A		–0.50	*<.001* ^e^
	Boxed distraction cost	N/A		–0.34	*.01* ^e^
	SAAT RT	N/A		–0.22	.10
	Spatial Span	N/A		0.24	.07
	Boxed RT	age, sex, edu, and BRT		–0.43	*.002* ^e^
	Boxed distraction cost	age, sex, edu, and BRT		–0.38	*.007* ^e^
	SAAT RT	age, sex, edu, and BRT		–0.16	.26
	Spatial Span	age, sex, edu, and BRT		0.18	.21

^a^SDMT: Symbol Digit Modalities Test.

^b^ACE: Adaptive Cognitive Evaluation.

^c^RT: reaction time.

^d^N/A: not applicable.

^e^*P* values in italic are significant.

^f^SAAT: Sustained Attention ACE Task.

^g^edu: years of education.

^h^BRT: basic reaction time.

^i^MS: multiple sclerosis.

**Table 3:** Results of Pearson correlation analyses between PASAT^a^ and ACE^b^ measures in participants with MS^c^ (N=53).

ACE measures	Covariates	R	*P* value
Boxed RT^d^	N/A^e^	–0.39	*.01* ^f^
Boxed distraction cost	N/A	–0.25	.07
SAAT^g^ RT	N/A	–0.03	.83
Spatial Span	N/A	0.29	*.03* ^f^
Boxed RT	age, sex, edu^h^, and BRT^i^	–0.40	*.01* ^f^
Boxed distraction cost	age, sex, edu, and BRT	–0.24	.09
SAAT RT	age, sex, edu, and BRT	–0.01	.92
Spatial Span	age, sex, edu, and BRT	0.34	*.02* ^f^

^a^PASAT: Paced Auditory Serial Addition Test.

^b^ACE: Adaptive Cognitive Evaluation.

^c^MS: multiple sclerosis.

^d^RT: reaction time.

^e^N/A: not applicable.

^f^*P* values in italic are significant.

^g^SAAT: Sustained Attention ACE Task.

^h^edu: years of education.

^i^BRT: basic reaction time.

### Group Differences in ACE

We then determined whether ACE modules can differentiate participants with MS with CI (SDMT z score<–1) and without CI (SDMT z score≥–1), as well as non-MS participants. To accomplish this, we conducted one-way ANOVA with the participant category as the independent variable for the Boxed RT, Boxed distraction cost, SAAT RT, and Spatial Span. We included age, sex, and years of education as covariates. The BRT was also included as a covariate since there was a significant difference in the BRT among the 3 groups (F (2,71)=3.96, *P*=.02), where the non-MS group showed a faster BRT (311.81 [14.50] ms) compared to both CI (362.86 [13.56] ms, *P*=.02) and non-CI (357.27 [12.34] ms, *P*=.02) participants with MS.

**Figure 5:**
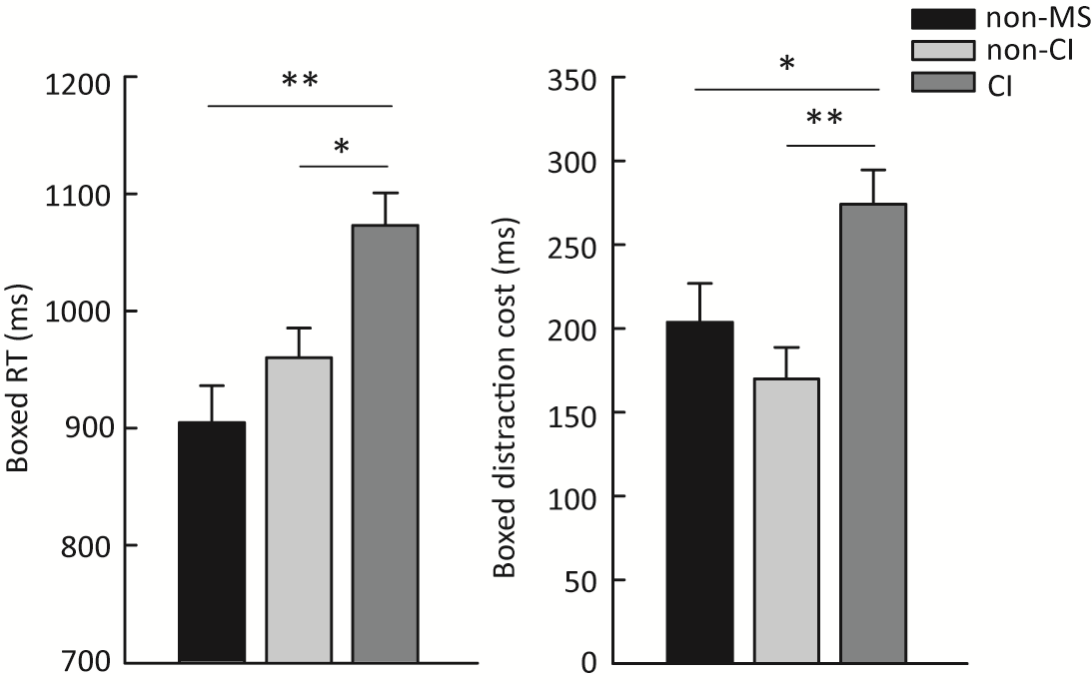
Group differences between CI, non-CI participants with MS and non-MS participants. Error bars represent SE.**P≦.001; *P≦.05. CI: cognitive impairment; MS: multiple sclerosis; RT: reaction time.

Significant group differences in the Boxed RT (F (2,66)=9.73, *P*<.001) and Boxed distraction cost (F (2,66)=7.40, *P*=.001) were revealed. Post hoc comparisons indicated a slower Boxed RT in CI (1072.72 [28.14] ms) compared to non-CI (959.89 [25.48] ms; *P*=.004) participants with MS as well as non-MS participants (904.86 [31.68] ms, *P*<.001). For Boxed distraction cost, CI participants showed a higher attention cost (274.35 [20.47] ms) when compared to non-CI participants (170.12 [18.53] ms, *P*<.001) and non-MS (203.84 [23.04] ms, *P*=.02) participants (Figure 5). Although no statistical differences in Boxed distraction cost were found between the non-CI and non-MS groups (*P*=.20), numerically, the distraction cost in the non-CI group was slightly lower than in the non-MS group. A lower RT cost may be attributed to 2 combinations of task performance: First, the same level of performance for the more challenging task condition but a worse performance in the easier task, and second, the same level of task performance for the easier task and a less performance drop in the more challenging task, indicating better cognitive ability as the performance is not affected much when the task becomes more difficult. Here, the numerical difference in Boxed distraction cost between non-CI and non-MS groups was more likely to be a result of worse performance in the easier task in the non-CI group (ie, slower RT in the 4-item condition) rather than a less performance change in the more challenging task (ie, faster RT in the 12-item condition). To understand this, we investigated RTs in 12-item and 4-item conditions in both groups. As expected, the 2 groups showed the same level of RT in the 12-item condition (non-CI vs non-MS: 1030.22 [24.09] ms vs 1022.23 [30.66] ms, *P*=0.84), but a slower RT was found in the non-CI group in the 4-item condition (non-CI vs non-MS: 854.37 [19.42] ms vs 814.89 [23.87] ms, *P*=0.23). The slower RT in the 4-item condition, to some extent, explained the numerically lower Boxed distraction cost in the non-CI group. No significant difference between the 3 groups was discovered for the SAAT RT (F (2,66)=0.42, *P*=.65) or Spatial Span (F (2,66)=0.62, *P*=.54). The results suggest that the ACE Boxed module can identify group-level differences between CI participants with MS, non-CI participants with MS, and non-MS participants.

## Discussion

### Principal Findings

In this study, we aimed to determine whether a digital cognitive assessment battery (ie, ACE) could be used to evaluate cognitive function in adults with MS with and without CI. We found a significant correlational association between ACE metrics and standard cognitive measures. When age, sex, years of education, and the BRT were considered as covariates, only correlations between the SDMT score and the Boxed RT as well as the Boxed distraction cost remained significant. Specifically, the ACE Boxed module, a task measuring visual search performance and attention [38], showed the strongest correlation with the SDMT and could identify group-level differences between adults with MS with and without CI, as well as adults without MS. Altogether, these results provide preliminary evidence that ACE, a tablet-based cognitive assessment battery, provides modules that could potentially serve as an unsupervised cognitive assessment for people with MS.

Correlational links between standard measures and ACE measures were discovered. Specifically, we noted significant correlations between higher SDMT scores and faster Boxed and SAAT RTs, lower Boxed distraction costs, and higher Spatial Span. The significant correlations between SDMT and Boxed measures in all participants (including both non-MS and MS) indicate that this ACE module is a potential cognitive assessment tool, and the results stand alone when only participants with MS are considered. Moreover, an exploratory simple linear regression analysis revealed a moderate R-squared value of 0.33, indicated that 33% of the total variation in the SDMT score can be explained by the Boxed RT. These findings partially support relative consequence theory [30-32], which postulates that a change in the information processing speed is a key deficit underlying cognitive dysfunction in MS [27-30]. However, a clear causal relationship between information processing speed and high-level cognitive performance cannot be concluded with the current results. Of note, when age, sex, years of education, and the BRT were considered as covariates, only correlations between the SDMT score and the Boxed RT and Boxed distraction cost remained significant. Boxed is a visual search task that requires participants to search for a specific target, filter out distractors, and provide a response. To some extent, the task structure and the domains of cognitive function being challenged are similar to those of the SDMT, in which participants are asked to substitute geometric symbols for numbers (make a response) while scanning a response key (search and filter out distractors). The similarity of the cognitive function subserving the 2 tasks may explain the consistent correlation between SDMT scores and Boxed performance. In contrast, the cognitive domains mainly being challenged in SAAT and Spatial Span are attention control and working memory, respectively, which are less similar to what is being challenged during an SDMT test. These different cognitive domains could have been affected differently by factors such as age, sex, and years of education, which could explain the marginally significant correlation revealed between the SDMT and performance in SAAT and Spatial Span when controlling for these factors.

Participants with MS with a higher PASAT score demonstrated a faster Boxed RT and better Spatial Span. PASAT is a test involving information processing, attention, and short-term maintenance and manipulation of information [36]. These associations were expected, as the Boxed module is designed to assess attention and information processing speed, and Spatial Span mainly contains the cognitive component of holding information in mind.

In addition to showing the correlational association between a subset of modules of ACE and standard cognitive measures, we further demonstrated that ACE could differentiate CI in adults with and without MS, as indicated by a significantly slower Boxed RT and the higher attention cost in CI compared to both non-CI participants with MS and non-MS participants. Of note, performance on SAAT and Spatial Span was not significantly different among the 3 groups. Compared to SAAT and Spatial Span, which only challenge 1 or 2 cognitive domains, Boxed is a task that is more complicated and requires more underlying cognitive resources to reach the task goal. Since there is large interindividual variability in the pattern of CI in MS [42,43], it is possible that a complex task requires more executive control and may be a more sensitive tool to capture cognitive dysfunction in participants with MS compared to tasks that challenge only 1 or 2 aspects of cognitive function. These results support our hypotheses that there are correlational links between performance in standard cognitive measures for MS and ACE modules. In addition, as a digital tool in assessing cognitive function in MS, at least 1 ACE module has the capacity to differentiate group-level differences among CI and non-CI MS participants and non-MS participants.

With the advances in digital technology, the assessment and treatment for people with MS have adopted digital platforms [44], which when used in the home can substantially improve accessibility to cognitive remediation programs. Recently, we demonstrated that in-game navigation features of an unsupervised, digital video game–based digital therapeutic could represent a novel and sensitive way to perform cognitive evaluations in MS [34]. The current results further support the use of a digital platform for cognitive assessment in MS. The built-in adaptive algorithms, which modulate task difficulty based on individual performance, reduce interindividual variability, which is usually a concern for cognitive assessments [45,46]. Since ACE is a self-guided digital assessment tool, it has the potential to be used in different settings (eg, at home or in the clinic). Future studies evaluating how ACE performance fluctuates during a day and whether the results would be affected by different testing environments are warranted. Moreover, since each ACE module is designed to challenge specific cognitive components, baseline ACE subtest scores could be useful to inform personalized cognitive training targets. For instance, for a participant with a low-grade score in Spatial Span and a high score in SAAT, the prescribed cognitive training approaches may place great emphasis on working memory rather than attentional control. Studies with a larger sample size or administering ACE as an outcome measure to capture the effect of a cognitive intervention are also needed to provide more information about how the ACE battery can reflect the patient’s and the caregiver’s real-life experience and to better translate the subtest scores to a meaningful treatment target.

### Limitations

Among the limitations of this study, the relatively small sample size and lack of multiple points of data collection at baseline made it difficult to draw a definitive conclusion with respect to the test validity and test-retest reliability of the ACE battery in people with MS. Related to this, given the predominately White participants in the study, particularly those with CI, the potential influence of racial and ethnicity on the results could not be fully excluded. Participants with severe CI were excluded from the study. Although the severity of CI can vary from mild to quite severe in MS patients, it has been reported that the majority (771/1014, 76.03%) of patients experience mild (340/771, 33.7%) to moderate (431/771, 42.7%) cognitive disturbance [47]. Since the application of the ACE program in clinical settings is still at an early stage, we planned to start with patients without severe CI to reduce the heterogeneity of the sample. Future studies with a broader range of CI are needed to investigate how the ACE tool performs when applied to participants with severe CI. Furthermore, the results of the exploratory simple linear regression analysis should be taken with caution, given the sample size does not have adequate power to provide a rigorous predictive model. Future studies with a larger sample size are warranted for a better predictive model development. Finally, experience with using digital tools may be confounding factors that can impact the results where participants with more experience in using digital tools may have performed better in this study. Future studies investigating digital assessments should control for participants’ skills in using digital devices.

### Conclusion

In summary, this study suggests that a tablet-based adaptive cognitive battery could be used to perform cognitive assessments in MS. As noted previously [20,34], the high adherence rate indicated that this remote, home-based health care strategy is well accepted by patients with MS, who may have limited access to cognitive assessment or treatment. Since CI poses major limitations to patients with MS, the current findings open up new paths to deploying digital cognitive tests for MS.

## Abbreviations

ACE: Adaptive Cognitive Evaluation
BRT: basic reaction time
CI: cognitive impairment
MS: multiple sclerosis
PASAT: Paced Auditory Serial Addition Test
RT: reaction time
SAAT: Sustained Attention ACE Task
SDMT: Symbol Digit Modalities Test
TOVA: Tests of Variables of Attention
UCSF: University of California, San Francisco
